# Artificial intelligence reshapes early oral cancer screening: from image recognition to risk prediction

**DOI:** 10.1097/JS9.0000000000003122

**Published:** 2025-08-06

**Authors:** Yan-Ling Zhang, Xin-Kui Zhang, Ting-Gang Fu

**Affiliations:** a Weifang Nursing Vocational college, Wei fang, Shan dong, China; b Weifang Dental Hospital, Wei fang, Shan Dong, China; c Department of Human Anatomy, Qilu Medical University, Zibo, Shan Dong, China

*Dear Editor,*

We appreciate the authors’ work in the recent article “Diagnostic accuracy of artificial intelligence assisted clinical imaging in the detection of oral potentially malignant disorders and oral cancer: a systematic review and meta-analysis.” This systematic review and meta-analysis comprehensively evaluates the application value of artificial intelligence (AI) in detecting oral potentially malignant disorders (OPMD) and oral cancers^[1]^. Through a rigorous literature screening process, our research team ultimately included 17 qualified studies for in-depth analysis. The results demonstrate that AI-assisted diagnosis exhibits outstanding performance metrics. Notably, among the three primary imaging modalities (clinical photography, optical coherence tomography [OCT], and autofluorescence imaging), clinical photography showed the highest diagnostic accuracy (DOR = 77.772) and possesses advantages of operational simplicity and cost-effectiveness, making it the most clinically promising screening method. Building upon this foundation, we offer the following insights for discussion.

Perspective 1 (transitioning from static image analysis to dynamic monitoring and multimodal data fusion): Current research predominantly focuses on AI analysis of static images, overlooking the dynamic evolutionary characteristics of oral lesions during disease progression^[2]^. In reality, many OPMD and early-stage oral cancers exhibit specific temporal patterns in morphology, color, and texture. Future AI systems should incorporate temporal image analysis capabilities, tracking changes in the same lesion across different time points to establish predictive models for assessing malignant transformation risk. Such dynamic monitoring methods could significantly improve the differentiation of “gray zone” lesions (those with ambiguous benign/malignant features).Multimodal data fusion represents another critically underexplored direction. Existing studies largely rely on single imaging techniques (e.g., using only clinical photography or OCT), whereas clinicians in practice typically synthesize findings from multiple examination methods. AI systems should be designed as integrated platforms capable of processing clinical photographs, OCT images, autofluorescence data, and even molecular biomarker test results simultaneously. Through deep learning-based multimodal fusion techniques, such systems could automatically weigh contributions from different data sources to generate more reliable diagnostic conclusions than any single modality could provide. For instance, while clinical photography excels at capturing surface morphological features, OCT reveals submucosal tissue architecture, and autofluorescence reflects tissue metabolic status – the combination of these complementary modalities could substantially enhance diagnostic accuracy.

Perspective 2 (developing human–AI collaborative diagnostic systems to outperform full automation): Current studies generally position AI systems as standalone diagnostic tools attempting to completely replace clinician judgment, an approach with fundamental limitations^[3]^. Oral lesion diagnosis is inherently a complex cognitive process involving pattern recognition, experiential judgment, and clinical context integration – capabilities difficult to replicate through fully automated systems. A more rational path involves constructing human–AI collaborative systems that leverage their respective strengths: AI excels at rapid processing of standardized images with consistency, while clinicians specialize in synthesizing multifaceted clinical information while considering individual variations. Such collaborative systems should incorporate several key features: First, beyond providing binary diagnostic outcomes (benign/malignant), AI should output interpretable supporting evidence, such as saliency heatmaps of lesion areas or similarity scores to prototypical cases, helping clinicians understand the rationale behind AI judgments. Second, the system requires an intuitive human–machine interface allowing clinicians to easily adjust AI parameters or override certain determinations, with these human interventions feeding back into the AI for continuous learning. Third, the system should record and analyze discrepancies between clinician and AI decisions, identifying lesion types where AI consistently underperforms for targeted algorithm improvement. This collaborative model proves particularly valuable for addressing AI systems’ current “long-tail problem” – those rare or atypical lesion types. Through real-time clinician feedback and corrections, the system can progressively expand its knowledge boundaries beyond the common patterns in training datasets. From an implementation perspective, pilot programs could begin in oral specialty hospitals where expert teams work alongside AI systems, gradually accumulating experience before expanding to primary care settings. The ultimate goal involves creating a new diagnostic ecosystem where AI enhances rather than replaces clinicians, jointly improving diagnostic accuracy and efficiency.

Perspective 3 (advancing personalized risk assessment models beyond lesion classification): Existing research primarily focuses on AI classification of oral lesions (e.g., normal/OPMD/cancer), neglecting the more clinically relevant need – predicting individual patients’ risk trajectories. In practice, what concerns clinicians and patients most isn’t the current lesion label but rather the probability and rate of future malignant transformation. AI technology should shift toward developing personalized risk assessment models that integrate imaging features with clinical parameters to provide dynamic risk predictions for each patient. This risk assessment model must consider multiple dimensions: First, lesion characteristics themselves – deep learning-extracted imaging biomarkers can correlate with known pathological features to establish malignancy risk scores. Second, patient-specific factors include genetic background (through simple genetic testing), lifestyle habits (tobacco/alcohol use), and immune status. Third, environmental factors like HPV infection status and geographic risk elements. Machine learning algorithms integrating these multisource data could generate personalized risk curves predicting malignant transformation probabilities at 1-year, 3-year, and 5-year intervals. Compared to traditional lesion classification, this prospective risk assessment represents a more precise medical paradigm, aligning with the overarching trend shifting oral cancer management from “diagnosis and treatment” to “prediction and prevention.” Although requiring more extensive data and complex algorithms, its clinical value would far surpass simple image classification systems, truly realizing AI’s transformative potential in oral cancer prevention and control.

This systematic review demonstrates AI’s excellent performance in oral cancer screening while highlighting current limitations including insufficient dataset standardization and lack of external validation. The proposed innovative directions **–** dynamic monitoring, human–AI collaboration, and risk prediction models **–** break through conventional research frameworks to suggest more forward-looking applications.

## Data Availability

Not applicable.
